# Treatment with bevacizumab and FOLFOXIRI in patients with advanced colorectal cancer: presentation of two novel trials (CHARTA and PERIMAX) and review of the literature

**DOI:** 10.1186/1471-2407-12-356

**Published:** 2012-08-16

**Authors:** Alexander Stein, Gabriel Glockzin, Andreas Wienke, Dirk Arnold, Thomas Edelmann, Bert Hildebrandt, Stephan Hollerbach, Gerald Illerhaus, Alfred Königsrainer, Michael Richter, Hans J Schlitt, Hans-Joachim Schmoll

**Affiliations:** 1 University Cancer Center Hamburg, University Medical Center Hamburg-Eppendorf, Hamburg, Germany; 2 Department of Surgery, University Medical Center Regensburg, Regensburg, Germany; 3 Department for Oncology/Hematology, Martin-Luther-University Halle, Ernst-Grube-Str. 40, 06120, Halle/Saale, Germany; 4 MedCenter Nordsachsen, Schkeuditz, Germany; 5 Charité Centrum für Tumormedizin, Campus Virchow-Klinikum, Berlin, Germany; 6 Department for Gastroenterology, Academic Teaching Hospital, Celle, Germany; 7 Department of Hematology/Oncology, Comprehensive Cancer Center University of Freiburg, Freiburg, Germany; 8 Department of Surgery, University Hospital of Tübingen, Tübingen, Germany; 9 Koordinierungszentrum Klinische Studien Halle, Martin-Luther-University, Halle, Germany

**Keywords:** FOLFOXIRI, Bevacizumab, Metastatic colorectal cancer, Resectable liver metastases

## Abstract

**Background:**

More than half of patients with colorectal cancer will develop metastatic disease either evident at the time of initial diagnosis or during their course of disease. Besides multidisciplinary management further treatment intensification is warranted to improve the still limited prognosis.

**Methods/design:**

In these two multi-centre, randomized phase II trials, conducted in Germany, 380 patients with R0-resectable colorectal liver metastases (PERIMAX) and with unresectable, metastatic colorectal cancer (CHARTA) will be recruited. Patients previously untreated for metastatic disease with either synchronous or metachronous metastases are randomly assigned in a 1:1 ratio to resection of colorectal liver metastases followed by postoperative FOLFOX for 6 months or perioperative FOLFOXIRI and bevacizumab for 3 months pre- and postoperative and resection (PERIMAX), or to induction chemotherapy with FOLFOX and bevacizumab +/− irinotecan for a maximum of 6 months followed by maintenance treatment with fluoropyrimidine and bevacizumab. The primary objective of these trials is to evaluate the feasibility and efficacy of FOLFOXIRI and bevacizumab in metastatic colorectal cancer. Primary endpoint is failure free survival rate at 18 months in the PERIMAX trial and progression free survival rate at 9 months in CHARTA. Secondary objectives include efficacy, safety and tolerability.

**Discussion:**

The CHARTA and PERIMAX trials are designed to evaluate the benefits and limitations of a highly active four-drug regimen in distinct treatment situations of metastatic CRC. Eligible patients are classified into resectable liver metastases to be randomized to perioperative treatment with FOLFOXIRI and bevacizumab or postoperative FOLFOX in the PERIMAX, or unresectable metastatic CRC to be randomized between FOLFOX and bevacizumab with or without irinotecan, stratified for clinical groups according to disease and patients’ characteristics in the CHARTA trial.

**Trial registration:**

Clinical trial identifier CHARTA: NCT01321957, PERIMAX: NCT01540435

## Background

Colorectal cancer (CRC) is the most frequently diagnosed cancer in Europe and one of the leading causes of cancer death worldwide [1,2]. About 20-25% of patients with CRC present with metastatic disease at time of diagnosis, and further 20-25% of patients will develop metastases after curative resection, of whom 20-30% present with liver metastases only [3,4]. Upfront stratification of patients with metastatic CRC (mCRC) into clinical groups according to clinical presentation and treatment aim by a multidisciplinary team is of utmost importance for the prognosis of the individual patient [5]. Clinical groups based on current guidelines and recommendations are displayed in Table 1[6,7]. Recent retrospective series demonstrated profound improvements in outcome of patients with colorectal cancer over the last 20 years due to advancements in chemotherapy and dramatic increase in patients undergoing liver resection for either primarily or secondarily resectable colorectal liver metastases (CLM), resulting in an overall survival (OS) comparable to that of stage III (UICC) colon cancer [8,9].

**Table 1:** Clinical groups for first-line treatment stratification

**group**	**clinical presentation**
0	clearly R0-resectable liver and/or lung metastases
1	unresectable liver and/or lung metastasis potentially resectable after downsizing, comorbidities allowing surgery
2	multiple metastasis, rapid progression, risk of rapid deterioration, unlikely to become resectable
3	never resectable and no symptoms or risk of deterioration

### Management of R0 resectable liver metastases

Resection of CLM is a potentially curative treatment, and reported 5-year survival rates are about 40% [10,11]. Further follow up after resection of liver metastases from colorectal cancer demonstrated long-term survival and finally cure for every fifth patient even in the presence of poor prognostic factors [12-15]. However, with the majority of patients relapsing after liver resection, either intrahepatic (about 70%), extrahepatic (about 50%), or both, further research is urgently warranted [16].

### Selection of patients for a potentially curative approach

The selection of patients for hepatic surgery is a controversial issue. The most commonly used scoring system for prediction of OS described by Fong and colleagues incorporates five risk factors: node-positive primary, disease-free interval <12 months, >1 lesion, size >5 cm, and CEA >200 ng/m. Patients with 5 risk factors had a 5 year OS rate (5-yOSR) of 14% with a median of 22 months compared to a 5-yOSR of 44% and a median of 51 months in case of one risk factor [14]. Recently, the international liver metastases survey, including data of about 4500 patients with preoperatively treated CLM, confirmed the poor prognosis of patients with lymph node positive primary, abnormal CEA levels, and a high number of CLM, although cut off value was >3 [15].

Whereas the estimation of survival is broadly accepted, criteria for resectability are far from being well and uniformly defined [17-21]. Generally, a post-resection remnant liver of less than 30%, unfavourable location of metastases, co-morbidities excluding major surgery, aggressive tumour biology, and/or presence of extrahepatic disease are regarded as limitations for liver surgery. However, liver resection should have the potential for complete macroscopic resection in curative intent [21].

### Perioperative treatment for resectable CLM

The EORTC 40983 trial accrued 364 patients to be randomized to two treatment arms: surgery alone or chemotherapy with 5-FU/LV and oxaliplatin (FOLFOX4 regimen), administered 3 months pre- and postoperatively. [22]. Patients had to be technically resectable (assessed by CT scan) with a maximum of 4 CLM and no prior treatment with chemotherapy. Despite favourable patients’ characteristics, with more than 50% bearing only a single CLM and about 80% with up to 2, respectively, and performance of surgery in “high quality” institutions, the reported 3 year progression free survival rates (3-yPFSR) were relatively poor. In the group of patients (n=171) receiving the planned chemotherapy and resection of CLM 3-yPFSR significantly increased from 33.2% (surgery alone) to 42.4% (HR 0.73, 95% CI 0.55 to 0.97). However, in the “intent to treat analyses”, the difference was not statistically significant due to ineligibility of 6% of patients (HR 0.79, 95% CI 0.62 to 1.02). No unusual toxicities occurred in the chemotherapy arm, and about 80% of patients completed the preoperative part. After resection, 76% of patients received postoperative treatment with the majority (52%; n=80) completing all 6 cycles.

Results of two single arm phase II trials using capecitabine, oxaliplatin and bevacizumab as neoadjuvant treatment in about 100 patients with CLM either clearly R0 resectable or unlikely R0 resectable with poor prognosis demonstrated feasibility of this regimen with an overall response rate (ORR) of 73-78% and a conversion rate of 40% (12 of 30) in the unlikely resectable group [23,24].

### Postoperative systemic treatment

Two randomized phase III trials have compared adjuvant systemic chemotherapy with 5FU/LV after resection of CLM to surgery alone, but both were closed prematurely due to slow accrual. By the time of closure, a small but statistically significant improvement in disease free survival (DFS) could be shown in the French trial, with a 5-year DFS rate of 26.7 vs. 33.5% (p=0.028), favouring the group with adjuvant treatment [25]. The ENG (EORTC/NCI-CTG/GIVIO) trial, still not fully published, showed a non-significant trend towards a prolongation of DFS (median 39 vs. 20 months; p=0.35) and an increase in overall survival (median 53 vs. 43 months; p = 0.39) [26]. The combined analysis of both trials (n=278 patients) showed a non-significant prolongation of DFS from 18.8 to 27.9 months (p = 0.058) and OS from 47.3 to 62.2 months (p=0.095) [27]. Both trials were using a (non-contemporary) 5-FU bolus regimen.

Intensification of postoperative treatment by 5-FU/LV plus irinotecan in a prematurely stopped, randomized phase III trial revealed no additional benefit compared to 5-FU/LV alone in terms of DFS (21.6 vs. 24.7 months, p=0.47) and OS (3-year rate of 71.6 vs. 72.7%, p=0.69) [28].

The Dutch HEPATICA trial, prematurely stopped after 74 randomized patients, indicated that postoperative treatment with capecitabine and oxaliplatin for 6 months and bevacizumab for 12 months might be beneficial with a trend in DFS rate at 2 years of 50 vs. 72% (p=0.074), although this trend needs to be carefully interpreted in regard of the known only transient benefit achieved with prolonged bevacizumab in stage II/III colon cancer [29-31].

### First line treatment for mCRC

Several first-line treatment options are currently available incorporating fluoropyrimidines, irinotecan, oxaliplatin, bevacizumab and EGFR antibodies (e.g. cetuximab and panitumumab) for KRAS wildtype patients [32-37].

### Bevacizumab based first line treatment for mCRC

A variety of trials and registry analyses evaluated the efficacy of bevacizumab-containing first-line regimens. The addition of bevacizumab to 5FU bolus and irinotecan regimen (IFL) significantly increased ORR and prolonged OS and PFS [38]. The NO16966 study was initially designed to prove non-inferiority of the XELOX regimen compared with FOLFOX-4, but the addition of bevacizumab in a 2x2 factorial design was amended after the above-mentioned results. Although ORR in this trial was not different between the bevacizumab and the placebo arms, potentially curative surgery was performed in 55 (9.6%) patients on bevacizumab vs. 38 (6.6%) on placebo (p=0.061) in the per protocol population. PFS was significantly increased in the bevacizumab group, 9.4 vs. 8.0 months in the placebo group (HR 0.83, 97.5% CI 0.72 to 0.95; p=0.0023). However, OS was 21.3 months in the bevacizumab group and 19.9 months in the placebo group (HR 0.89, 97.5% CI 0.76 to 1.03; p=0.077) [34]. Several observational phase IIIb/IV trials (e.g., First BEAT, BriTE) confirmed the safety profile of bevacizumab in first-line mCRC patients receiving a variety of chemotherapy regimens, namely FOLFOX, XELOX, FOLFIRI, or capecitabine [39,40].

### Four drug regimens for mCRC

After the negative trials combining a chemo-doublet with bevacizumab and EGFR antibodies, four drug regimens focused on a chemo-triplet in combination with a targeted drug [41,42]. Several chemo-triplet schedules with 5FU/LV or capecitabine, irinotecan and oxaliplatin were evaluated with the Italian FOLFOXIRI demonstrating the most efficacious and reliable data [37,43-46]. The FOLFOXIRI regimen by Falcone et al. significantly increased ORR (34 vs. 60%, p<0.0001), PFS (6.9 vs. 9.8 months, p=0.0006), and OS (16.7 vs. 22.6 months, p=0.032) compared to FOLFIRI in a phase III trial with 244 patients. Furthermore, the FOLFOXIRI regimen resulted in an increased R0 secondary resection rate (6% vs. 15%, p=0.033, among all 244 patients; and 12 vs. 36%, p=0.017 among patients with CLM only) [37]. Administration of second line does not seem to be relevantly impaired by first line FOLFOXIRI according to recently presented data demonstrating feasibility and efficacy of second line treatment with either oxaliplatin or irinotecan based doublet (38%), rechallenge with FOLFOXIRI (24%) or fluoropyrimidine with or without mitomycin [47]. Retreatment with FOLFOXIRI was associated with significantly prolonged survival.

Several single arm phase II trials with different 5FU/LV, irinotecan and oxaliplatin schedules were performed in combination with either bevacizumab or cetuximab resulting in high ORR of 75-82% [48-53]. However, in the absence of comparative data, toxicity profile (especially grade 3/4 diarrhoea) seems to favour bevacizumab-containing regimens (14-28%) compared to 36-93% observed with cetuximab combinations even after dose reduction of chemotherapy.

In a recently published single arm phase II trial, an ORR of 77% in all patients and 80% in the liver-only population (n=30) was achieved with the combination of FOLFOXIRI and bevacizumab. R0 resections were performed in 40% (n=12) of the liver-only population. Disease control rate was 100%. PFS and OS were 13.1 and 30.9 months, respectively. Treatment was well tolerated with the major grade 3/4 toxicity being neutropenia in 49% of patients (compared to 50% with FOLFOXIRI alone), which was well manageable with secondary G-CSF prophylaxis leading to only one case of febrile neutropenia. Further G3/4 toxicities were diarrhoea in 14%, hypertension in 11%, and asthenia as well as deep vein thrombosis in 7% of patients [52]. Similar results could be achieved in another 5FU/LV, irinotecan and oxaliplatin regimen (weekly alternating “Poker” schedule) with bevacizumab [49]. Furthermore, early safety results of a phase III trial by the Italian group comparing FOLFIRI and bevacizumab with or without oxaliplatin revealed no unexpected toxicities in the first 100 randomized patients [54].

## Methods/design

### Study design

Both trials are multicentre, open labelled, prospective, randomized phase II studies. The study protocols were approved by the local ethics committees, and were also subject to authorization by the competent authority (BfArM for CHARTA and PEI for PERIMAX) as mandatory by federal law. All participants have to provide written informed consent. The trials were assigned the EudraCT numbers 2010-022162-27 for CHARTA and 2010-023575-25 for PERIMAX and are registered at ClinicalTrials.gov (CHARTA: NCT01321957, PERIMAX: NCT01540435).

### Study objectives and endpoints

The primary objective of the CHARTA study is to evaluate the efficacy of FOLFOXIRI and bevacizumab compared to FOLFOX and bevacizumab in patients with initially unresectable metastatic colorectal cancer. Secondary objectives are safety and tolerability of the treatment, efficacy in terms of secondary resectability, prognostic value of stratification into clinical groups, and validity of allocation to these groups and the exploratory question whether the addition of irinotecan might be more effective in terms of response and survival in patient groups to be determined by angiogenic marker profiles, potentially indicating different sensitivity to bevacizumab. Primary endpoint is PFS rate at 9 months; secondary endpoints include PFS, OS, ORR (according to RECIST v1.1), secondary resection rate, toxicity (according to NCI-CTCAE v4.0) and quality of life (according to EORTC QLQ-C30 and modules CR29 and CIPN20) [55]. PFS is defined as time from randomization to date of first observed progression or death (without reintroduction).

The primary objective of the PERIMAX study is to evaluate the efficacy of FOLFOX for 6 months postoperatively compared to FOLFOXIRI and bevacizumab for three months pre- and three months postoperatively for primarily resectable liver metastases from colorectal cancer. Secondary objectives are safety and tolerability of the treatment as well as efficacy in terms of survival. Primary endpoint is failure- free survival (FFS) rate at 18 months. Failure will be defined as macroscopically incomplete resection (R2), local or distant recurrence or death from any cause. Secondary endpoints are PFS, FFS, OS, achievability of macroscopically complete resection (R0/1), ORR (according to RECIST v1.1) after preoperative treatment (arm B), toxicity (according to NCI-CTCAE v 4.0), perioperative morbidity, quality of life (according to EORTC QLQ-C30 and module LMC21), and survival according to molecular or clinico-pathological factors.

### Patient selection

Patients with histologically confirmed diagnosis of mCRC can be included in the CHARTA-PERIMAX trials, independent of synchronous or metachronous metastases, although in case of synchronous disease in the PERIMAX trial the primary tumour needs to be asymptomatic and clearly R0 resectable. Inclusion into the PERIMAX trial is limited to patients with R0 resectable CLM as judged by the treating physician and thus excludes patients with extrahepatic disease. Measurable disease according to RECIST, adequate ECOG-PS (≤2 in CHARTA, although ECOG-PS 2, only if tumour related; ≤1 in PERIMAX), age≥18 (upper limit in the PERIMAX at 75 years of age), prior adjuvant treatment completed either at least 6 months before inclusion in the CHARTA, or at least 12 months for oxaliplatin-based treatment in the PERIMAX, are further selection criteria. Prerequisite laboratory values for both trials are absolute neutrophil counts ≥1.5 x 10^9^/L, platelets ≥100 x10^9^/L, haemoglobin ≥9 g/dl or 5.59 mmol/l, INR <1.5 and aPTT < 1.5 Upper Limit of Normal (ULN) for patients not receiving therapeutic anticoagulation (use of full dose anticoagulants is allowed as long as the INR or aPTT is within therapeutic limits and the patient has been on a stable dose for anticoagulants for at least two weeks at the time of registration), serum transaminases (AST & ALT) ≤ 2.5 x ULN (in case of liver metastases < 5 x ULN), total bilirubin ≤ 1.5 x ULN, and creatinine ≤ 1.5 x ULN. Further selection criteria are based on current standard criteria (e.g. no pregnancy or breast feeding, signed and dated consent form) and the contraindications of the used agents (e.g. severe thrombosis or bleeding, major surgery within 28 days, chronic diarrhoea or significant peripheral neuropathy).

### Treatment schedule

**Figure 1:**
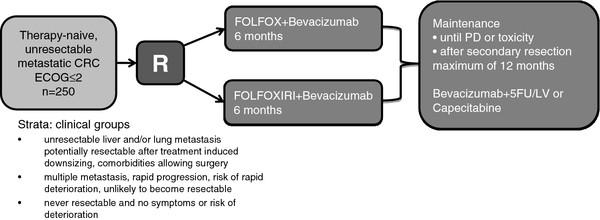
overview CHARTA trial.

**Figure 2:**
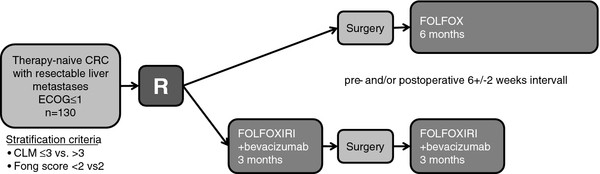
overview PERIMAX trial.

Treatment schedules for CHARTA and PERIMAX are summarized in Figures 1 and 2, respectively. Patients will randomly be assigned in a 1:1 ratio stratified for the clinical groups one to three mentioned in Table 1 in the CHARTA trial and for number of CLM ≤3 vs >3 and Fong score <2 vs. ≥2 in the PERIMAX trial.

### CHARTA trial

Induction chemotherapy with a modified FOLFOX with oxaliplatin at a dose of 85 mg/m^2^ iv over two hours (day 1), LV at a dose of 200 mg/m^2^ iv over two hours (day 1) and 5-FU at a dose of 3200 mg/m^2^ iv over 48 hours (day 1–3) and bevacizumab at a dose of 5 mg/kg iv over 30 to 90 min (day 1) with or without irinotecan at a dose of 165 mg/m^2^ iv over one hour (day 1) in a biweekly schedule will be administered followed by maintenance with either 5-FU/LV and bevacizumab (same dosage and schedule as above) or capecitabine at a dose of 1600 mg/m^2^ in two doses po day 1 to 14 and bevacizumab at a dose of 7.5 mg/kg iv over 30 to 90 min (day 1) every three weeks (choice of 5-FU or capecitabine is at the discretion of the investigator). Treatment with FOLFOX and bevacizumab +/− irinotecan will be administered until progression, intolerable toxicity, and secondary resection or for a maximum of 12 cycles (6 months). After 6 months of treatment and/or no progression patients will continue with a maintenance regimen with bevacizumab and a fluoropyrimidine for up to 12 months in the absence of progression or intolerable toxicity. Maximum treatment duration is 18 months (6 months of FOLFOX and bevacizumab +/− irinotecan followed by 12 months of maintenance). In case of secondary resection (at any time point) treatment should be resumed 4–8 weeks postoperatively for a total of 6 months FOLFOX and bevacizumab +/− irinotecan (pre- and postoperative treatment), followed by maintenance treatment for total treatment duration of up to 12 months in the absence of progression or intolerable toxicity. Reintroduction of oxaliplatin +/− irinotecan or restart of treatment in case of progressive disease during maintenance or complete break after 18 months of treatment is at the investigators discretion.

### PERIMAX trial

The same regimens as in the CHARTA trial will be administered, although the postoperative treatment in arm A is FOLFOX without bevacizumab. In arm A FOLFOX will be administered for 12 cycles (6 months) postoperatively starting 6 +/−2 weeks after surgery. In the perioperative arm (B) FOLFOXIRI and bevacizumab will be administered for 6 cycles (last cycle without bevacizumab) (3 months) preoperatively, followed by liver surgery within 6+/−2 weeks after last treatment FOLFOXIRI (without bevacizumab), followed by 6 cycles (3 months) 6+/−2 weeks postoperatively. Bevacizumab should be withheld for at least 5 weeks before conducting surgery.

The first cycle of FOLFOXIRI in both trials can be administered with 75% of dosage, if no diarrhoea or other significant toxicities ≥ grade 2 occurs, following cycles should be administered in full dosage. Dose reduction and escalation is at the discretion of the investigator. Preoperative dose modifications should be maintained for postoperative treatment in the PERIMAX trial. If a patient was treated with full dose preoperatively, dose reduction to 75% for the first cycle and subsequent escalation might be applied similar to preoperative treatment. Doses of drugs will be reduced in case of undue toxicity. Treatment will be delayed until adequate recovery from toxicity.

### Assessments/follow up

Tolerability will be evaluated at every visit including physical examination, vital signs, clinical laboratory profile, and adverse events, which will be graded according to NCI-CTCAE v.4.0 and correlated to the administered treatment or performed study procedure. Treatment related Serious Adverse Events (SAE) considered possibly, probably or definitely related to treatment, will be determined.

In both trials the investigator will assess tumour response according to RECIST v1.1 and CEA (optional CA 19–9). CT and/or MRI scans will be independently reviewed e.g. for resectability (both trials) and allocation to the clinical groups (CHARTA). In the CHARTA trial imaging of all sites of disease will be performed at baseline and every 4 cycles (8 weeks) thereafter for the first 6 months (induction treatment) and afterwards every 3 months (during and after maintenance) until progression.

Radiological imaging in the PERIMAX trial (CT-scan of the chest, CT or MRI-scan of the abdomen) should be done at baseline and/or preoperatively, postoperatively (prior to the start of postoperative treatment) and afterwards every three months for two years and every 6 month thereafter in both arms. In the preoperatively treated arm, an additional radiological imaging after the 3^rd^ cycle (6 weeks) should be done to avoid clinically complete response. In case of CEA elevation without progressive disease defined by RECIST, further examinations must be performed searching for local recurrence or metastatic progression of disease. Diagnosis of recurrence could either be made by radiological imaging or by positive cytology or biopsy. After progression, patients will be followed every 3 months for disease status, protracted toxicities, further treatment, and survival.

Quality of life will be assessed together with tumour assessment using the EORTC QLQ-C30, and the modules CR29 and CIPN20 in the CHARTA and the module LMC21 in the PERIMAX.

Formalin fixed and paraffin embedded (FFPE) tumour tissue obtained at baseline (if available) and serum obtained at baseline and before the 3^rd^ and the 7^th^ cycle will be evaluated for KRAS and BRAF-status (FFPE) and angiogenic markers (FFPE and serum) in the CHARTA trial. In addition to KRAS and BRAF in PERIMAX evaluation of pathohistological response rate and further analyses are planned, thus either paraffin embedded or preferably fresh frozen tissue of primary tumour and hepatic metastases should be obtained in patients consenting. Furthermore, serum obtained at baseline, during preoperative treatment, postoperatively and at follow up will be evaluated for circulating tumour DNA.

### Statistical considerations and sample size estimation

#### CHARTA trial

First line therapy with FOLFOX and bevacizumab displays a median PFS of 9,4 months [34], leading to a PFS Rate at 9 months (PFSR@9) of approximately 55%. The four-drug combination treatment should produce a PFSR@9 of at least 71.3%. The risk of estimating the four-drug combination treatment as active although the PFSR@9 is less than 71.3% should be 10%. The risk of rejecting the therapy although the PFSR@9 is more than 71.3% should be 20%, which leads to a power of 80%. The planned number of patients to answer this question in a two-sided (continuity corrected) Chi-square test will be 120 and with a dropout rate of 4% the number to be included will be 125 patients per arm. Analysis will be performed by logistic regression to account for the stratification.

#### PERIMAX trial

Perioperative chemotherapy with 5-FU/LV with or without oxaliplatin in a prognostic favourable group of patients with up to 4 CLM led to a FFS rate at 18 months (FFSR@18) of 50-65% [22,25]. However, in the trial by Portier et al. solely patients after R0 resection were included, whereas in the EORTC study 51% and 27% of patients had one or two CLM, respectively. In this trial, patients with unlimited number of CLM, fulfilling the local judgement for resectability will be included. Therefore, FFSR@18 is expected to be 40% with current treatment strategies (postoperative FOLFOX). The investigational treatment (perioperative FOLFOXIRI and bevacizumab) should produce a FFSR@18 of at least 60%, to be regarded as promising for further evaluation and of major clinical relevance. The trial should achieve 80% power to detect differences between the treatments and keeping the type I error level below 10% in a one-sided (continuity corrected) Chi-square test. The planned number of patients to answer this question with a drop out rate of about 14% will be 65 patients per arm. The trial uses a randomized parallel arm phase II design.

### Quality assurance/safety

Patient data are collected in a case report form at the data centre of the clinical research organisation (Koordinierungszentrum für Klinische Studien Halle). Consistency checks will be performed on newly entered forms and queries issued in case of inconsistencies. On-site monitoring will be performed adapted according to the site accrual. Data safety monitoring boards will review the data from both trials on a regular basis.

## Discussion

The CHARTA and PERIMAX trials are designed to evaluate the benefits and limitations of a highly active four-drug regimen in different treatment situations of metastatic CRC with nearly 200 patients randomized to an experimental treatment with FOLFOXIRI and bevacizumab vs. what is considered a standard approach.

Perioperative treatment in resectable CLM, especially its sequence (peri- vs. postoperative treatment) and intensity, is still a matter of debate. The PERIMAX trial design represents the agreement on a multidisciplinary trial by surgical and medical oncologists in Germany and reflects the ongoing discussion about timing of perioperative chemotherapy. In regard of the high recurrence rate after resection with curative intent and the results of four drug combinations including bevacizumab in mCRC, further intensification of the perioperative treatment to improve patients’ prognosis by increasing the rate of long-term survivors seems to be a feasible option. Despite the lack of data for FOLFOX for purely postoperative treatment after resection of CLM, this regimen was chosen in regard of the beneficial trend for 5FU/LV in this setting and the data obtained in stage III disease with oxaliplatin [27,56,57]. Furthermore, this treatment approach is widely used for patients receiving upfront resection of CLM, and is considered a ‘current standard’ for this patient group. However, the PERIMAX trial has several variables (sequence and intensity) and is therefore of an exploratory nature focusing on the efficacy and feasibility of perioperative FOLFOXIRI and bevacizumab in this setting. The randomized design was chosen to collect data albeit limited by the low number of patients on the comparative efficacy in terms of survival in order to reduce the bias that is associated with a comparison to historical controls, which are only available for 5FU/LV in this setting [58]. Number of CLM with a cut-off value of 3 was added to the widely accepted stratification according to the Fong score. Although already part of the Fong score, this cut off value has recently demonstrated high prognostic value [15]. The composite endpoint FFS was chosen to adapt to the specific situation in this trial with half of the patients being disease-free within the first months after randomization, compared to the other half receiving 3 months of preoperative treatment. In order to ensure comparability with available data, e.g. EORTC 40983 (PFS), and to gain information about tolerability and QoL with the four-drug regimen secondary endpoints include these parameters. Perioperative treatment was limited to 6 months, as no conclusive data about prolonged treatment is currently available [30]. The trial also offers the unique opportunity of collecting tissue from liver metastases and primary tumours either untreated or treated. Therefore, fresh frozen and paraffin embedded tissue will be collected and stored for further analyses.

The CHARTA trial will evaluate the efficacy of a maximal intensive first line regimen compared to a standard first line regimen in patients with unresectable metastatic CRC. The intensification of first line treatment is believed to increase the rate of patients amenable for a secondary curative approach and even if surgery might not be an option this treatment approach could result in improved long-term disease control. Especially for clinical group 2 and 3, if amenable for intensive first line treatment, disease control after induction chemotherapy could result in long intervals of either low toxic maintenance therapy or complete treatment discontinuation and might thus be a reasonable long-term approach. Furthermore, the magnitude and point in time of tumour response has demonstrated significant impact on prognosis, underlining the need for an intensive first line regimen inducing early tumour shrinkage [59]. Eligible patients will be stratified according to clinical groups in order to balance the arms especially for potentially secondary resectable patients. PFS-rate at 9 months was chosen as primary endpoint to gain information about the comparative efficacy in terms of a survival endpoint, taking into account the inclusion of all metastatic and not only potentially resectable patients. Treatment duration is limited to a maximum of 6 months FOLFOX and bevacizumab +/− irinotecan, followed by up to 12 months of maintenance with fluoropyrimidine and bevacizumab. According to current clinical standard oxaliplatin should be discontinued at least after 6 months of treatment due to significant cumulative side effects, particularly peripheral neuropathy. None of the available trials with FOLFOXIRI with or without bevacizumab continued treatment beyond 12 cycles (6 months), as tolerability will be significantly impaired and achievement of secondary resectability with prolonged treatment beyond 6 months seems unlikely [37,52]. In the CELIM trial for example median time to resection or exploration was 5.1 months, with a median number of 8 treatment cycles until intervention [60]. Current data support the use of maintenance treatment after first line chemotherapy, though the choice of regimen is not yet clear [61-64]. However, results of ongoing trials (e.g. AIO 0207 and CAIRO3) might help clarifying this question and potentially the choice of maintenance regimen as well (biological +/− fluoropyrimidine. In regard of the higher intensity of the four-drug combination not only in terms of response but as well in terms of toxicity QoL evaluation is performed during the trial.

With overall 380 previously untreated metastatic CRC patients classified for resectability, stratified for clinical grouping and randomized, the CHARTA and PERIMAX trials will give further information about the benefit and tolerability of FOLFOXIRI and bevacizumab in these distinct disease settings.

## Abbreviations

5-FU, 5-fluorouracil; 5-yOSR, 5 year overall survival rate; AE, Adverse event; ALT, Alanin-aminotransferase; AST, Aspartat-aminotransferase; aPTT, Activated partial thromboplastin time; BfArM, Bundesinstitut für Arzneimittel und Medizinprodukte; BRAF, V-raf murine sarcoma viral oncogene homolog B1; CA 19–9, Carbohydrate antigen 19–9; CEA, Carcinoembryonic antigen; CLM, Colorectal liver metastases; CT, Computed tomography; DFS, Disease free survival; DNA, Deoxyribonucleic acid; ECOG-PS, Eastern cooperative oncology group – performance status; EGFR, Epidermal growth factor receptor; EORTC, European organisation for research and treatment of cancer; EudraCT, European Clinical Trials Database; FFS, Failure free survival; FFSR@18, Failure free survival rate at 18 months; FOLFIRI, 5-FU/LV and irinotecan; FOLFOX, 5-FU/LV and oxaliplatin; FOLFOXIRI, 5-FU/LV, oxaliplatin and irinotecan; G, Grade; G-CSF, Granulocyte colony-stimulating factor; HR, Hazard ratio; IFL, 5FU bolus and irinotecan regimen; INR, International Normalized Ratio; Iv, Intravenous; KRAS, Kirsten rat sarcoma viral oncogene homolog; LV, Leucovorin; mCRC, Metastatic colorectal cancer; min, Minutes; MRI, Magnetic resonance imaging; N, Number of patients; NCI-CTCAE, National Cancer Institute common terminology criteria for adverse events; ORR, Overall response rate; OS, Overall survival; P, P-value; PEI, Paul Ehrlich Institut; PFS, Progression free survival; PFSR@9, Progression free survival rate at 9 months; Po, Per os; RECIST, Response evaluation criteria in solid tumours; SAE, Severe adverse event; QoL, Quality of life; QLQ, Quality of life questionnaire; UICC, Union internationale contre le cancer; ULN, Upper Limit of Normal; V, Version; Vs, Versus; XELOX, Capecitabine and oxaliplatin.

## Competing interests

Both trials are funded by Roche. AS has received honoraria from Roche and Merck KGaA. HJS has received research funding and honoraria from Roche. H-JS and DA have received honoraria and research funding from Roche and Merck KGaA and honoraria from Amgen. BH has received honoraria and research funding from Amgen, Roche and Merck KGaA. AW, AK, GG, MR and TE declare that there is no conflict of interest.

## Authors’ contributions

AS and GG prepared the manuscript. HJS and H-JS are the coordinating investigators for the two trials and participated in the preparation of manuscript and study protocols. AS wrote both study protocols, GG participated in the PERIMAX protocol. DA coordinates the translational part of the CHARTA trial. AW performed the statistical analysis for both trials and participated in drafting of the manuscript. MR coordinates both trials as clinical research associate. TE, BH, SH, GI, AK participate in patient recruitment in both trials. All authors read and approved the final manuscript.

## Pre-publication history

The pre-publication history for this paper can be accessed here:

http://www.biomedcentral.com/1471-2407/12/356/prepub

## References

[B1] Jemal A Bray F Center MM Ferlay J Ward E Forman D Global cancer statistics CA Cancer J Clin 2011 61 69 90 10.3322/caac.20107 21296855

[B2] Ferlay J Shin HR Bray F Forman D Mathers C Parkin DM Estimates of worldwide burden of cancer in 2008: GLOBOCAN 2008 Int J Cancer 2010 127 12 2893 2917 10.1002/ijc.25516 21351269

[B3] van der Pool AE Damhuis RA Ijzermans JN de Wilt JH Eggermont AM Kranse R Verhoef C Trends in incidence, treatment and survival of patients with stage IV colorectal cancer: a population-based series Colorectal Dis 2012 14 56 61 10.1111/j.1463-1318.2010.02539.x 21176063

[B4] Mantke R Schmidt U Wolff S Kube R Lippert H Incidence of synchronous liver metastases in patients with colorectal cancer in relationship to clinico-pathologic characteristics. Results of a German prospective multicentre observational study Eur J Surg Oncol 2012 38 259 265 10.1016/j.ejso.2011.12.013 22209659

[B5] Schmoll HJ Sargent D Single agent fluorouracil for first-line treatment of advanced colorectal cancer as standard? Lancet 2007 370 105 107 10.1016/S0140-6736(07)61062-9 17630019

[B6] Van Cutsem E Dicato M Arber N Berlin J Cervantes A Ciardiello F De Gramont A Diaz-Rubio E Ducreux M Geva R Molecular markers and biological targeted therapies in metastatic colorectal cancer: expert opinion and recommendations derived from the 11th ESMO/World Congress on Gastrointestinal Cancer, Barcelona, 2009 Ann Oncol 2010 21 uppl 6 vi1 10 20534623 10.1093/annonc/mdq273

[B7] Schmiegel W Pox C Arnold D Porschen R Rodel C Reinacher-Schick A Colorectal carcinoma: the management of polyps, (neo)adjuvant therapy, and the treatment of metastases Dtsch Arztebl Int 2009 106 843 848 20062582 10.3238/arztebl.2009.0843 PMC2803611

[B8] Kopetz S Chang GJ Overman MJ Eng C Sargent DJ Larson DW Grothey A Vauthey JN Nagorney DM McWilliams RR Improved survival in metastatic colorectal cancer is associated with adoption of hepatic resection and improved chemotherapy J Clin Oncol 2009 27 3677 3683 10.1200/JCO.2008.20.5278 19470929 PMC2720081

[B9] Morris EJ Forman D Thomas JD Quirke P Taylor EF Fairley L Cottier B Poston G Surgical management and outcomes of colorectal cancer liver metastases Br J Surg 2010 97 1110 1118 10.1002/bjs.7032 20632280

[B10] Jonas S Thelen A Benckert C Spinelli A Sammain S Neumann U Rudolph B Neuhaus P Extended resections of liver metastases from colorectal cancer World J Surg 2007 31 511 521 10.1007/s00268-006-0140-3 17308854

[B11] Wei AC Greig PD Grant D Taylor B Langer B Gallinger S Survival after hepatic resection for colorectal metastases: a 10-year experience Ann Surg Oncol 2006 13 668 676 10.1245/ASO.2006.05.039 16523369

[B12] Tomlinson JS Jarnagin WR DeMatteo RP Fong Y Kornprat P Gonen M Kemeny N Brennan MF Blumgart LH D’Angelica M Actual 10-year survival after resection of colorectal liver metastases defines cure J Clin Oncol 2007 25 4575 4580 10.1200/JCO.2007.11.0833 17925551

[B13] Scheele J Stang R Altendorf-Hofmann A Paul M Resection of colorectal liver metastases World J Surg 1995 19 59 71 10.1007/BF00316981 7740812

[B14] Fong Y Fortner J Sun RL Brennan MF Blumgart LH Clinical score for predicting recurrence after hepatic resection for metastatic colorectal cancer: analysis of 1001 consecutive cases Ann Surg 1999 230 309 318 discussion 318–321 10.1097/00000658-199909000-00004 10493478 PMC1420876

[B15] Adam R Barroso E Impact of the type and modalities of preoperative chemotherapy on the outcome of liver resection for colorectal metastases J Clin Oncol 2011 29 abstr 3519

[B16] de Jong MC Pulitano C Ribero D Strub J Mentha G Schulick RD Choti MA Aldrighetti L Capussotti L Pawlik TM Rates and patterns of recurrence following curative intent surgery for colorectal liver metastasis: an international multi-institutional analysis of 1669 patients Ann Surg 2009 250 440 448 19730175 10.1097/SLA.0b013e3181b4539b

[B17] Nagashima I Takada T Nagawa H Muto T Okinaga K Proposal of a new and simple staging system of colorectal liver metastasis World J Gastroenterol 2006 12 6961 6965 17109517 10.3748/wjg.v12.i43.6961 PMC4087339

[B18] Poston GJ Figueras J Giuliante F Nuzzo G Sobrero AF Gigot JF Nordlinger B Adam R Gruenberger T Choti MA Urgent need for a new staging system in advanced colorectal cancer J Clin Oncol 2008 26 4828 4833 10.1200/JCO.2008.17.6453 18711170

[B19] Van Cutsem E Nordlinger B Adam R Kohne CH Pozzo C Poston G Ychou M Rougier P Towards a pan-European consensus on the treatment of patients with colorectal liver metastases Eur J Cancer 2006 42 2212 2221 10.1016/j.ejca.2006.04.012 16904315

[B20] Yamaguchi T Mori T Takahashi K Matsumoto H Miyamoto H Kato T A new classification system for liver metastases from colorectal cancer in Japanese multicenter analysis Hepatogastroenterology 2008 55 173 178 18507101

[B21] Nordlinger B Van Cutsem E Gruenberger T Glimelius B Poston G Rougier P Sobrero A Ychou M Combination of surgery and chemotherapy and the role of targeted agents in the treatment of patients with colorectal liver metastases: recommendations from an expert panel Ann Oncol 2009 20 985 992 10.1093/annonc/mdn735 19153115

[B22] Nordlinger B Sorbye H Glimelius B Poston GJ Schlag PM Rougier P Bechstein WO Primrose JN Walpole ET Finch-Jones M Perioperative chemotherapy with FOLFOX4 and surgery versus surgery alone for resectable liver metastases from colorectal cancer (EORTC Intergroup trial 40983): a randomised controlled trial Lancet 2008 371 1007 1016 10.1016/S0140-6736(08)60455-9 18358928 PMC2277487

[B23] Wong R Cunningham D Barbachano Y Saffery C Valle J Hickish T Mudan S Brown G Khan A Wotherspoon A A multicentre study of capecitabine, oxaliplatin plus bevacizumab as perioperative treatment of patients with poor-risk colorectal liver-only metastases not selected for upfront resection Ann Oncol 2011 22 2042 2048 10.1093/annonc/mdq714 21285134

[B24] Gruenberger B Tamandl D Schueller J Scheithauer W Zielinski C Herbst F Gruenberger T Bevacizumab, capecitabine, and oxaliplatin as neoadjuvant therapy for patients with potentially curable metastatic colorectal cancer J Clin Oncol 2008 26 1830 1835 10.1200/JCO.2007.13.7679 18398148

[B25] Portier G Elias D Bouche O Rougier P Bosset JF Saric J Belghiti J Piedbois P Guimbaud R Nordlinger B Multicenter randomized trial of adjuvant fluorouracil and folinic acid compared with surgery alone after resection of colorectal liver metastases: FFCD ACHBTH AURC 9002 trial J Clin Oncol 2006 24 4976 4982 10.1200/JCO.2006.06.8353 17075115

[B26] Langer B Bleiberg H Labianca R Fluorouracil (FU) plus l-leucovorin (l-LV) versus observation after potentially curative resection of liver or lung metastases from colorectal cancer (CRC): results of the ENG (EORTC/NCIC CTG/GIVIO) randomized trial Proc Am Soc Clin Oncol 2002 21 abstr 592 2002

[B27] Mitry E Fields AL Bleiberg H Labianca R Portier G Tu D Nitti D Torri V Elias D O’Callaghan C Adjuvant chemotherapy after potentially curative resection of metastases from colorectal cancer: a pooled analysis of two randomized trials J Clin Oncol 2008 26 4906 4911 10.1200/JCO.2008.17.3781 18794541

[B28] Ychou M Hohenberger W Thezenas S Navarro M Maurel J Bokemeyer C Shacham-Shmueli E Rivera F Kwok-Keung Choi C Santoro A A randomized phase III study comparing adjuvant 5-fluorouracil/folinic acid with FOLFIRI in patients following complete resection of liver metastases from colorectal cancer Ann Oncol 2009 20 1964 1970 10.1093/annonc/mdp236 19567451

[B29] Andre T Van Cutsem E A multinational, randomized phase III study of bevacizumab (Bev) with FOLFOX4 or XELOX versus FOLFOX4 alone as adjuvant treatment for colon cancer (CC): Subgroup analyses from the AVANT trial J Clin Oncol 2011 29 abstr 3509

[B30] Voest EE Snoeren N A randomized two-arm phase III study to investigate bevacizumab in combination with capecitabine plus oxaliplatin (CAPOX) versus CAPOX alone in post radical resection of patients with liver metastases of colorectal cancer J Clin Oncol 2011 29 abstr 3565 10.1186/1471-2407-10-545 PMC2958953 20937118

[B31] Allegra CJ Yothers G O’Connell MJ Sharif S Petrelli NJ Colangelo LH Atkins JN Seay TE Fehrenbacher L Goldberg RM Phase III trial assessing bevacizumab in stages II and III carcinoma of the colon: results of NSABP protocol C-08 J Clin Oncol 2011 29 11 16 10.1200/JCO.2010.30.0855 20940184 PMC3055856

[B32] Douillard JY Siena S Cassidy J Tabernero J Burkes R Barugel M Humblet Y Bodoky G Cunningham D Jassem J Randomized, phase III trial of panitumumab with infusional fluorouracil, leucovorin, and oxaliplatin (FOLFOX4) versus FOLFOX4 alone as first-line treatment in patients with previously untreated metastatic colorectal cancer: the PRIME study J Clin Oncol 2010 28 4697 4705 10.1200/JCO.2009.27.4860 20921465

[B33] Van Cutsem E Kohne CH Lang I Folprecht G Nowacki MP Cascinu S Shchepotin I Maurel J Cunningham D Tejpar S Cetuximab Plus Irinotecan, Fluorouracil, and Leucovorin As First-Line Treatment for Metastatic Colorectal Cancer: Updated Analysis of Overall Survival According to Tumor KRAS and BRAF Mutation Status J Clin Oncol 2011 29 2011 2019 10.1200/JCO.2010.33.5091 21502544

[B34] Saltz LB Clarke S Diaz-Rubio E Scheithauer W Figer A Wong R Koski S Lichinitser M Yang TS Rivera F Bevacizumab in combination with oxaliplatin-based chemotherapy as first-line therapy in metastatic colorectal cancer: a randomized phase III study J Clin Oncol 2008 26 2013 2019 10.1200/JCO.2007.14.9930 18421054

[B35] Maughan TS Adams RA Smith CG Meade AM Seymour MT Wilson RH Idziaszczyk S Harris R Fisher D Kenny SL Addition of cetuximab to oxaliplatin-based first-line combination chemotherapy for treatment of advanced colorectal cancer: results of the randomised phase 3 MRC COIN trial Lancet 2011 377 2103 2114 10.1016/S0140-6736(11)60613-2 21641636 PMC3159415

[B36] Bokemeyer C Bondarenko I Hartmann JT de Braud F Schuch G Zubel A Celik I Schlichting M Koralewski P Efficacy according to biomarker status of cetuximab plus FOLFOX-4 as first-line treatment for metastatic colorectal cancer: the OPUS study Ann Oncol 2011 22 1535 1546 10.1093/annonc/mdq632 21228335

[B37] Falcone A Ricci S Brunetti I Pfanner E Allegrini G Barbara C Crino L Benedetti G Evangelista W Fanchini L Phase III trial of infusional fluorouracil, leucovorin, oxaliplatin, and irinotecan (FOLFOXIRI) compared with infusional fluorouracil, leucovorin, and irinotecan (FOLFIRI) as first-line treatment for metastatic colorectal cancer: the Gruppo Oncologico Nord Ovest J Clin Oncol 2007 25 1670 1676 10.1200/JCO.2006.09.0928 17470860

[B38] Hurwitz H Fehrenbacher L Novotny W Cartwright T Hainsworth J Heim W Berlin J Baron A Griffing S Holmgren E Bevacizumab plus irinotecan, fluorouracil, and leucovorin for metastatic colorectal cancer N Engl J Med 2004 350 2335 2342 10.1056/NEJMoa032691 15175435

[B39] Van Cutsem E Rivera F Berry S Kretzschmar A Michael M DiBartolomeo M Mazier MA Canon JL Georgoulias V Peeters M Safety and efficacy of first-line bevacizumab with FOLFOX, XELOX, FOLFIRI and fluoropyrimidines in metastatic colorectal cancer: the BEAT study Ann Oncol 2009 20 1842 1847 10.1093/annonc/mdp233 19406901

[B40] Grothey A Sugrue MM Purdie DM Dong W Sargent D Hedrick E Kozloff M Bevacizumab beyond first progression is associated with prolonged overall survival in metastatic colorectal cancer: results from a large observational cohort study (BRiTE) J Clin Oncol 2008 26 5326 5334 10.1200/JCO.2008.16.3212 18854571

[B41] Hecht JR Mitchell E Chidiac T Scroggin C Hagenstad C Spigel D Marshall J Cohn A McCollum D Stella P A randomized phase IIIB trial of chemotherapy, bevacizumab, and panitumumab compared with chemotherapy and bevacizumab alone for metastatic colorectal cancer J Clin Oncol 2009 27 672 680 10.1200/JCO.2008.19.8135 19114685

[B42] Tol J Koopman M Cats A Rodenburg CJ Creemers GJ Schrama JG Erdkamp FL Vos AH van Groeningen CJ Sinnige HA Chemotherapy, bevacizumab, and cetuximab in metastatic colorectal cancer N Engl J Med 2009 360 563 572 10.1056/NEJMoa0808268 19196673

[B43] Bajetta E Celio L Ferrario E Di Bartolomeo M Denaro A Dotti K Mancin M Bajetta R Colombo A Pusceddu S Capecitabine plus oxaliplatin and irinotecan regimen every other week: a phase I/II study in first-line treatment of metastatic colorectal cancer Ann Oncol 2007 18 1810 1816 10.1093/annonc/mdm347 17823385

[B44] Ychou M Viret F Kramar A Desseigne F Mitry E Guimbaud R Delpero JR Rivoire M Quenet F Portier G Nordlinger B Tritherapy with fluorouracil/leucovorin, irinotecan and oxaliplatin (FOLFIRINOX): a phase II study in colorectal cancer patients with non-resectable liver metastases Cancer Chemother Pharmacol 2008 62 195 201 10.1007/s00280-007-0588-3 17901955

[B45] Souglakos J Androulakis N Syrigos K Polyzos A Ziras N Athanasiadis A Kakolyris S Tsousis S Kouroussis C Vamvakas L FOLFOXIRI (folinic acid, 5-fluorouracil, oxaliplatin and irinotecan) vs FOLFIRI (folinic acid, 5-fluorouracil and irinotecan) as first-line treatment in metastatic colorectal cancer (MCC): a multicentre randomised phase III trial from the Hellenic Oncology Research Group (HORG) Br J Cancer 2006 94 798 805 10.1038/sj.bjc.6603011 16508637 PMC2361370

[B46] Zarate R Rodriguez J Bandres E Patino-Garcia A Ponz-Sarvise M Viudez A Ramirez N Bitarte N Chopitea A Gacia-Foncillas J Oxaliplatin, irinotecan and capecitabine as first-line therapy in metastatic colorectal cancer (mCRC): a dose-finding study and pharmacogenomic analysis Br J Cancer 2010 102 987 994 10.1038/sj.bjc.6605595 20216541 PMC2844042

[B47] Fornaro L Vasile E Masi G Loupakis F Baldi GG Allegrini G Salvatore L Cremolini C Cupini S Cortesi E Outcome of Second-Line Treatment After First-Line Chemotherapy With the GONO FOLFOXIRI Regimen Clin Colorectal Cancer 2012 11 71 76 10.1016/j.clcc.2011.06.013 21903485

[B48] Assenat E Desseigne F Thezenas S Viret F Mineur L Kramar A Samalin E Portales F Bibeau F Crapez-Lopez E Cetuximab Plus FOLFIRINOX (ERBIRINOX) as first-line treatment for unresectable metastatic colorectal cancer: a phase II trial Oncologist 2011 16 11 1557 1564 10.1634/theoncologist.2011-0141 22016477 PMC3233290

[B49] Bruera G Santomaggio A Cannita K Baldi PL Tudini M De Galitiis F Mancini M Marchetti P Antonucci A Ficorella C Ricevuto E “Poker” association of weekly alternating 5-fluorouracil, irinotecan, bevacizumab and oxaliplatin (FIr-B/FOx) in first line treatment of metastatic colorectal cancer: a phase II study BMC Cancer 2010 10 567 10.1186/1471-2407-10-567 20958992 PMC2972284

[B50] Trarbach T Schuette J Dose escalating study of 5-FU/folinic acid (FA)/oxaliplatin/irinotecan (FOLFOXIRI) and cetuximab in first-line therapy of patients with metastatic colorectal cancer J Clin Oncol 2009 27 abstr e15025

[B51] Ychou M Desseigne F Preliminary results of a multicentre phase II trial evaluating cetuximab in combination with FOLFIRINOX (LV5FU + Irinotecan + Oxaliplatin) as first line treatment of metastatic colorectal cancer (mCRC) patients Gastrointestinal Cancers Symposium 2009 2009 (Abstract-No: 295 abstr. 450

[B52] Masi G Loupakis F Salvatore L Fornaro L Cremolini C Cupini S Ciarlo A Del Monte F Cortesi E Amoroso D Bevacizumab with FOLFOXIRI (irinotecan, oxaliplatin, fluorouracil, and folinate) as first-line treatment for metastatic colorectal cancer: a phase 2 trial Lancet Oncol 2010 11 845 852 10.1016/S1470-2045(10)70175-3 20702138

[B53] Garufi C Torsello A Tumolo S Ettorre GM Zeuli M Campanella C Vennarecci G Mottolese M Sperduti I Cognetti F Cetuximab plus chronomodulated irinotecan, 5-fluorouracil, leucovorin and oxaliplatin as neoadjuvant chemotherapy in colorectal liver metastases: POCHER trial Br J Cancer 2010 103 1542 1547 10.1038/sj.bjc.6605940 20959822 PMC2990583

[B54] Falcone A Loupakis F FOLFOXIRI plus bevacizumab (BV) versus FOLFIRI plus BV as first-line treatment of metastatic colorectal cancer (MCRC): Preliminary safety results of the phase III randomized TRIBE study by the Gruppo Oncologico Nord-Ovest (GONO) J Clin Oncol 2010 28 abstr 3543

[B55] Eisenhauer EA Therasse P Bogaerts J Schwartz LH Sargent D Ford R Dancey J Arbuck S Gwyther S Mooney M New response evaluation criteria in solid tumours: revised RECIST guideline (version 1.1) Eur J Cancer 2009 45 228 247 10.1016/j.ejca.2008.10.026 19097774

[B56] Andre T Boni C Navarro M Tabernero J Hickish T Topham C Bonetti A Clingan P Bridgewater J Rivera F de Gramont A Improved overall survival with oxaliplatin, fluorouracil, and leucovorin as adjuvant treatment in stage II or III colon cancer in the MOSAIC trial J Clin Oncol 2009 27 3109 3116 10.1200/JCO.2008.20.6771 19451431

[B57] Haller DG Tabernero J Maroun J de Braud F Price T Van Cutsem E Hill M Gilberg F Rittweger K Schmoll HJ Capecitabine Plus Oxaliplatin Compared With Fluorouracil and Folinic Acid As Adjuvant Therapy for Stage III Colon Cancer J Clin Oncol 2011 29 1465 1471 10.1200/JCO.2010.33.6297 21383294

[B58] Ratain MJ Sargent DJ Optimising the design of phase II oncology trials: the importance of randomisation Eur J Cancer 2009 45 275 280 10.1016/j.ejca.2008.10.029 19059773

[B59] Piessevaux H Schlichting M Early tumor shrinkage for the prediction of efficacy of cetuximab in metastatic colorectal cancer: analysis from the CRYSTAL study Ann Oncol 2010 21 abstr. 596

[B60] Folprecht G Gruenberger T Bechstein WO Raab HR Lordick F Hartmann JT Lang H Frilling A Stoehlmacher J Weitz J Tumour response and secondary resectability of colorectal liver metastases following neoadjuvant chemotherapy with cetuximab: the CELIM randomised phase 2 trial Lancet Oncol 2010 11 38 47 10.1016/S1470-2045(09)70330-4 19942479

[B61] Wasan H Adams RA Intermittent chemotherapy (CT) plus continuous or intermittent cetuximab (C) in the first-line treatment of advanced colorectal cancer (aCRC): Results of the two-arm phase II randomized MRC COIN-b trial J Clin Oncol 2012 30 abstr 536

[B62] Adams RA Meade AM Seymour MT Wilson RH Madi A Fisher D Kenny SL Kay E Hodgkinson E Pope M Intermittent versus continuous oxaliplatin and fluoropyrimidine combination chemotherapy for first-line treatment of advanced colorectal cancer: results of the randomised phase 3 MRC COIN trial Lancet Oncol 2011 12 642 653 10.1016/S1470-2045(11)70102-4 21641867 PMC3159416

[B63] Chibaudel B Maindrault-Goebel F Lledo G Mineur L Andre T Bennamoun M Mabro M Artru P Carola E Flesch M Can chemotherapy be discontinued in unresectable metastatic colorectal cancer? The GERCOR OPTIMOX2 Study J Clin Oncol 2009 27 5727 5733 10.1200/JCO.2009.23.4344 19786657

[B64] Diaz-Rubio E Gomez-Espana A Massuti B Sastre J Abad A Valladares M Rivera F Safont MJ Martinez de Prado P Gallen M First-Line XELOX Plus Bevacizumab Followed by XELOX Plus Bevacizumab or Single-Agent Bevacizumab as Maintenance Therapy in Patients with Metastatic Colorectal Cancer: The Phase III MACRO TTD Study Oncologist 2012 17 15 25 10.1634/theoncologist.2011-0249 22234633 PMC3267817

